# Coronal and axial alignment relationship in Caucasian patients with osteoarthritis of the knee

**DOI:** 10.1038/s41598-021-87483-6

**Published:** 2021-04-09

**Authors:** Vicente J. León-Muñoz, Silvio Manca, Mirian López-López, Francisco Martínez-Martínez, Fernando Santonja-Medina

**Affiliations:** 1 Department of Orthopaedic Surgery and Traumatology, Hospital Clínico Universitario Virgen de la Arrixaca, Ctra. Madrid-Cartagena, s/n, El Palmar, 30120 Murcia, Spain; 2 Patient Matched Technology Department, Medacta International SA, Strada Regina, Castel San Pietro, 6874 Ticino, Switzerland; 3 Subdirección General de Tecnologías de la Información, Servicio Murciano de Salud, Avenida Central, 7, Edificio Habitamia, Espinardo, 30100 Murcia, Spain; 4 Department of Surgery, Pediatrics and Obstetrics and Gynecology (Faculty of Medicine), University of Murcia, Campus de Espinardo, Calle Campus Universitario, s/n, 30100 Murcia, Spain

**Keywords:** Anatomy, Medical research, Rheumatology

## Abstract

Individualized pre-operative assessment of the patterns of the lower extremity anatomy and deformities in patients undergoing total knee arthroplasty seems essential for a successful surgery. In the present study, we investigated the relationship among the coronal alignment and the rotational profile of the lower extremities in the Caucasian population with end-stage knee osteoarthritis. We conducted a prospective study of 385 knees that underwent a pre-operative three-dimensional computed tomography-based model. The lower extremity alignment was determined (mechanical tibiofemoral or hip-knee-ankle angle, supplementary angle of the femoral lateral distal angle, and proximal medial tibial angle). For each case, the femoral distal rotation (condylar twist angle), the femoral proximal version, and the tibial torsion were determined. As the coronal alignment changed from varus to valgus, the femoral external rotation increased (r = 0.217; p < 0.0005). As the coronal alignment changed from varus to valgus, the external tibial torsion increased (r = 0.248; p < 0.0005). No correlation was found between the global coronal alignment and the femoral version. The present study demonstrates a linear relationship between the coronal alignment and the rotational geometry of the distal femur. This correlation also occurs with the tibial torsion. Perhaps outcomes of total knee arthroplasty surgery might be improved by addressing these deformities as well.

## Introduction

The best method of total knee arthroplasty (TKA) alignment remains controversial^1^. Many authors advocate the traditional neutral mechanical alignment concepts (components perpendicular to the mechanical axis of each bone)^2,3^. Others postulate a personalized TKA implantation to generate a more physiological prosthetic knee, restoring the pre-arthritic anatomy of an individual patient (by aligning the components with the three kinematic axes of the native knee)^4,5^.

Despite this interesting and necessary discussion, pre-operative assessment of the patterns of the lower extremity anatomy and deformities in patients undergoing TKA seems essential for a successful surgery. Previous studies have reported rotation of the femoral component as an influential factor in patellofemoral and tibiofemoral kinematics and the dynamic alignment of the lower limb after TKA^6^. The relationship between the coronal and rotational alignment of the femur in both the healthy knee and the osteoarthritic (OA) knee has been published, focusing on the rotational deformity of the distal femur only^7–10^. The analysis has also been extended to the relationship among the coronal alignment and the rotational profile of the lower extremities, including rotational geometry of the distal femur, femoral anteversion, and tibial torsion in the Asian population^11^. Several studies have shown knee anatomic differences between Asian and Caucasian populations^10,12–16^. The present study aimed to investigate the relationship among the coronal alignment and the rotational profile of the lower extremities, including rotational geometry of the distal femur, femoral anteversion, and tibial torsion in the Caucasian population with knee OA. The authors hypothesized a significant correlation between the coronal alignment and the rotational variables.

## Methods

We conducted a prospective, observational, and monocentric study (level II of evidence) of 385 knees in 322 Caucasian patients (staged bilateral or simultaneous bilateral TKA was performed in 63 patients). They underwent a pre-operative computed tomography scan (CT-scan) assessment of the lower limb between January 2012 and May 2019. The CT-scan study was part of the design procedure for MYKNEE patient-specific TKA instrumentation (Medacta International SA, Castel San Pietro, Switzerland). All cases presented endstage knee OA. The mean age was 70.5 ± 8.2 years. The mean height was 160.8 ± 7.4 cm, and the average weight was 75 ± 8.9 kg (mean BMI was 29.1 ± 3.7 kg/m^2^). Females represented 60.6% of the total number of patients (195 women and 127 men). The exclusion criteria for the study were histories of bone fracture, bone abnormality, or surgery at the affected limb (hip, knee, or ankle).

Image acquisition has not differed from that standardized for surgery planning and that we have used in other studies^17^. Somatom Scope scanner (Siemens Healthcare GmbH, Erlangen, Germany) was used. Images were taken with the patient lying supine at isocenter in the gantry with the leg of interest in complete extension. The acquisition has consisted of three separate short spiral axial scans: hip, affected knee, and ankle, without moving the patient. Each acquisition was centered and zoomed accurately to ensure that the field of view (FoV) maximizes the region of interest. Scans were acquired in slices of a minimum of 512 × 512 pixels. The thickness of a single slice was 2 mm for the hip and ankle, and 0.6 mm for the knee, with a maximum FoV of 200 mm. We have adjusted the voltage peak to 130 kV and the X-ray tube current to 60 mA. The average effective dose of radiation per CT-scan (hip, knee, and ankle) was 0.4 mSv^17^.

Like other studies^9^, CT-scans were analyzed in a CT-based computer modeling software program MYPLANNER (Medacta International, Castel San Pietro, Switzerland) to generate three-dimensional virtual models. One trained Medacta planning engineer performed all measurements up to one decimal precision (0.1°)^9^. The virtual model's measurement was based on mean determinations (computer-automated algorithm) of the reference points on which to perform the angular measurements (in the same way as when employing computer-assisted surgery and mapping the different anatomical locations) and not on single points^17^.

For the definition of the different anatomical landmarks, we have used identical criteria to those used in another study by our group^17^. The center of the hip was defined as the center of the sphere that best approximates the femoral head. The center of the distal femur was identified as the middle of the intercondylar notch (most distal point of the trochlea). The center of the tibia was located at the midpoint of a line drawn between medial and lateral intercondylar eminences. The center of the ankle was defined as the mean point between the most protruding part of the medial malleolus and the most protruding distal part (tip of the fibula) of the lateral malleolus. The lower extremity alignment was determined using the mechanical tibiofemoral or hip-knee-ankle (HKA) angle measured with CT-scan in non-weight-bearing conditions^17^. We have used the same definition of angles and alignment criteria as in other studies^11,17^. HKA was defined as the angle between the femoral and the tibial mechanical axes on the medial side. The femoral mechanical axis was defined as the line connecting the center of the hip and the center of the intercondylar notch. The tibial mechanical axis was defined as the line connecting the point between the medial and lateral intercondylar eminences and the center of the ankle. Concerning HKA, values below 177° represent varus, and values above 183° represent valgus alignment. The femoral articular axis (line among the most distal points of the femoral condyles) and the tibial articular axis (line among the deepest points on the tibial plateau) were also determined. The supplementary angle of the femoral lateral distal angle (sFLDA) was defined as the angle on the medial side between the mechanical axis of the femur and the distal femoral articular axis. The proximal medial tibial angle (PMTA) was defined as the angle on the medial side between the mechanical axis of the tibia and the proximal tibial articular axis. Concerning sFLDA and PMTA, values lower than 90° indicate varus alignment, and values higher than 90° indicate valgus alignment relative to the mechanical axis of femur and tibia^17^.

**Figure 1:**
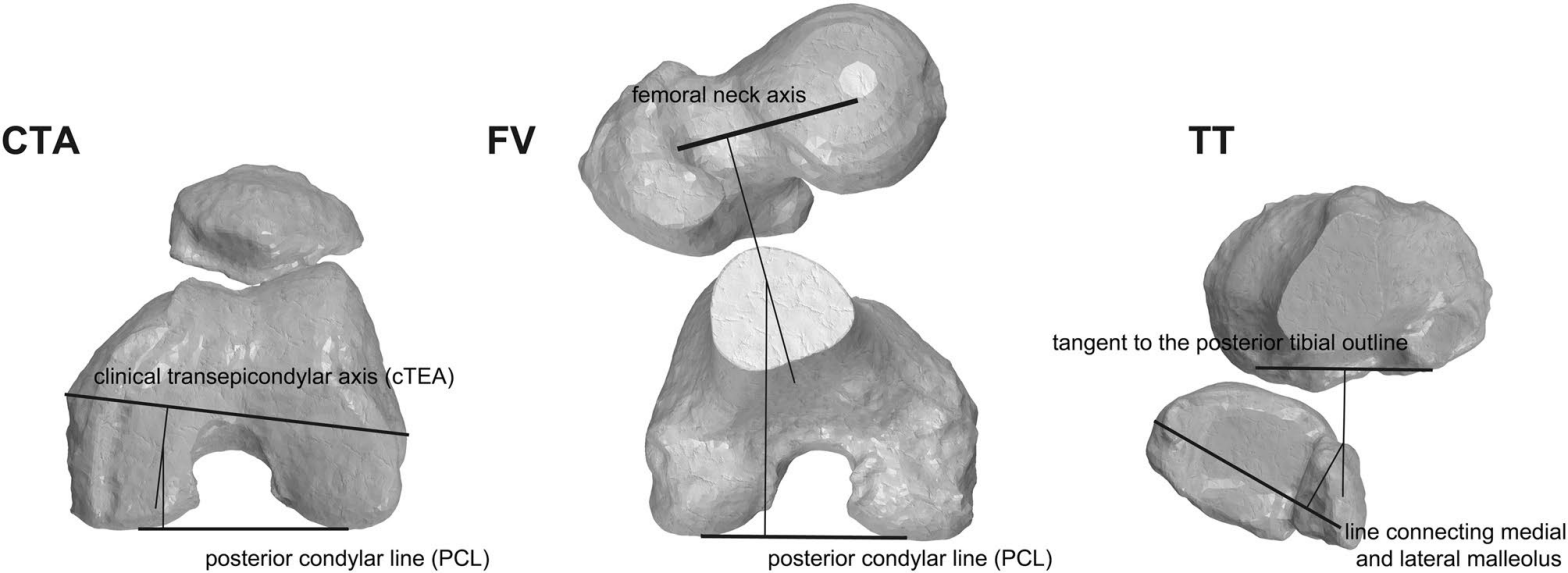
Anatomical references on the 3D virtual model to determine the rotational characteristics of the limb. *CTA* condylar twist angle: angle between the posterior condylar line and the clinical or anatomical transepicondylar axis. *FV* femoral version: angle between the femoral neck axis and the posterior condylar line. *TT* tibial torsion: angle between the line connecting the posterior cortices of the proximal tibial condyles and the line connecting the most prominent points of the medial and lateral malleolus.

In the axial plane (Fig. 1), the anatomical references necessary to characterize the lower limb rotationally were recorded: the femoral neck axis (a line connecting the center of the femoral head and the femoral anatomical axis passing through the center of the neck section), the posterior condylar line (PCL) (the line connecting the most posterior margins of both lateral and medial posterior condyles), the clinical or anatomical transepicondylar axis (cTEA) (a line connecting the tip of medial and lateral epicondylar prominences of the femur), the line connecting the posterior cortices of the proximal tibial condyles (a tangent to the posterior tibial outline) and the line connecting both medial and lateral malleolus (the most prominent points).

The external femoral rotation was measured by the condylar twist angle (CTA), first described by Yoshioka et al.^18^. The CTA is defined as the angle between the PCL and the cTEA. Femoral Version (FV) was measured as the angle formed between the femoral neck axis and the PCL^19^. Positive values indicate anteversion, antetorsion, or anterotation (the femoral neck axis inclines anterior to the PCL), and negative values indicate retroversion, retrotorsion, or retrorotation (the femoral neck axis inclines posterior to the PCL). The Tibial Torsion (TT) was determined by the angle formed between the line connecting the posterior cortices of the proximal tibial condyles and the line connecting the most prominent points of the medial and lateral malleolus^20^. Positive values represented a relative external rotation of the distal tibia to the proximal, whereas negative values represented a relative internal rotation.

Statistical analysis was performed using the Statistical Package for the Social Sciences (SPSS), version 25 for Windows (SPSS, Inc., Chicago, Illinois, USA). Patients' demographics were summarized using descriptive statistics. Given the size of the sample (385) and according to the central limit theorem, we assumed as normal the distribution variables. Pearson's correlation analysis was used to determine the statistical significance of the relationship between the coronal alignment and the rotational profile of the lower extremity^11^. Linear regression analysis for assessment of the variables that influence the coronal alignment was also performed. In addition (as in Chang's analysis^11^), patients were divided into three groups according to the HKA: HKA < 177° = varus group (n = 251), HKA 177° to 183° = neutral group (n = 81) and HKA > 183° = valgus group (n = 53). Patient demographic data are shown in Table 1. One-way analysis of variance (ANOVA) was conducted to determine the statistical significance of the differences among the three groups in terms of the axial alignment. A post hoc analysis was performed using the Bonferroni method. All differences were considered significant at a probability level of 95% (p < 0.05).

**Table 1:** Patient demographic data of the varus, neutral and valgus group.

	Varus group	Neutral group	Valgus group	p-value
Age (years)	71.1 (7.6)	70.8 (8.7)	67.4 (9.3)	0.01
Gender (female:male)	130:121	54:27	40:13	0.001
Side (right:left)	125:126	40:41	37:16	0.025
Height (cm)	161.5 (7.6)	160.1 (6)	158.4 (7.6)	0.014
Weight (kg)	75.2 (9)	74.6 (8.7)	74.3 (9)	0.725
BMI (kg/cm^2^)	28.9 (3.7)	29.2 (3.7)	29.7 (3.4)	0.401

Data are presented as the mean and (standard deviation). *BMI* body mass index.

### Ethics committee information

The ethics committee of the Hospital General Universitario José María Morales Meseguer has approved the study (CPVLM012019CIEST16/19). Informed consent from participants was waived by the institutional ethics committee, given the anonymization procedure of patient data. Our study followed the ethical standards of the World Medical Association Declaration of Helsinki, as revised in 2013.

## Results

**Figure 2:**
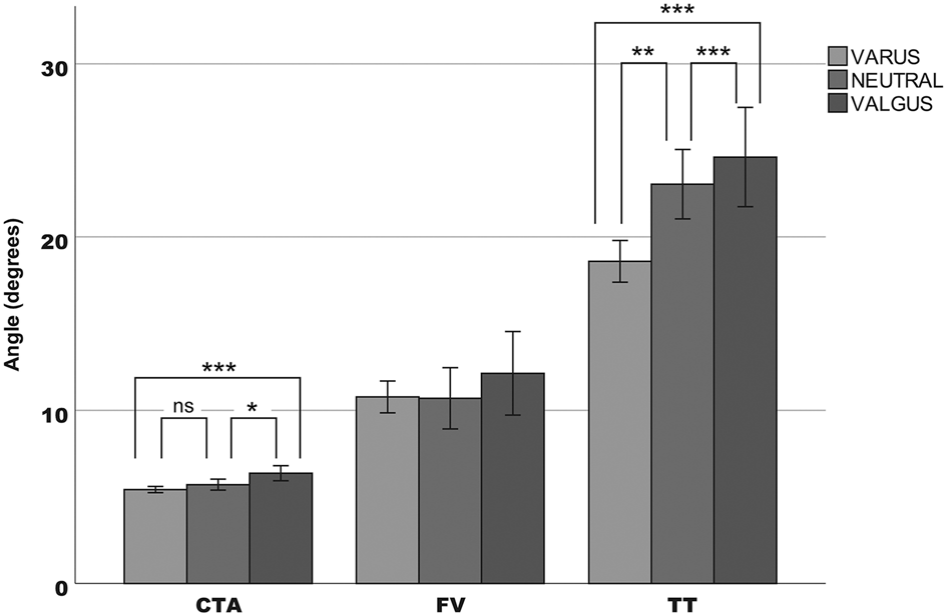
Comparison of rotational parameters among the groups divided by coronal alignment. The external Femoral Rotation and the external Tibial Torsion increased as the coronal alignment changed from varus to valgus. *CTA* condylar twist angle, *FV* Femoral Version, *TT* Tibial Torsion. *p < 0.05; **p < 0.001; ***p < 0.0005. *ns* no significant.

**Figure 3:**
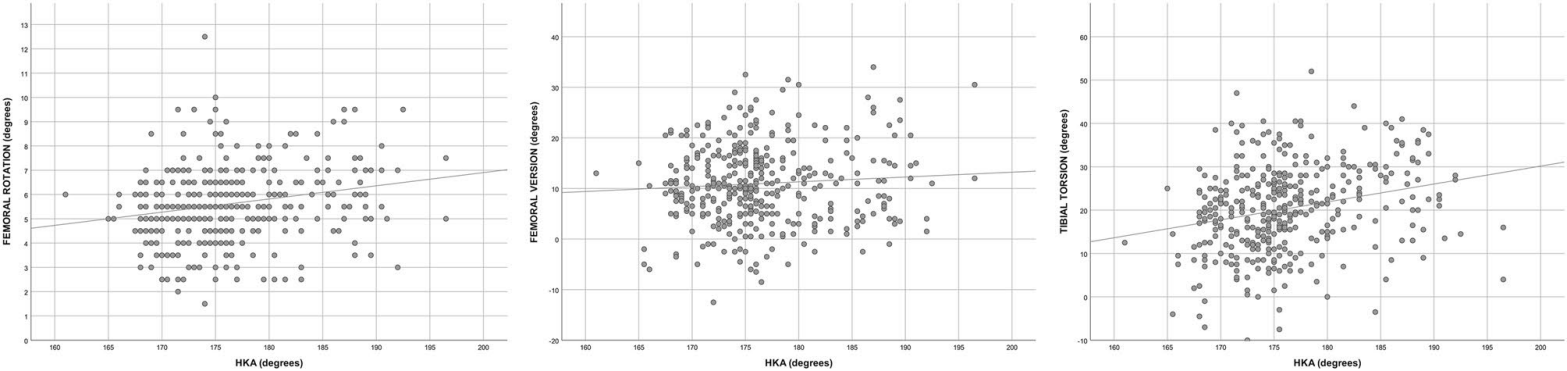
Scatter plots of the rotational alignment with regard to the coronal alignment of the limb. HKA, hip-knee-ankle angle. For the Femoral Version, positive values represent anteversion and negative values retroversion. For the Tibial Torsion, positive values represent external tibial torsion and negative, internal tibial torsion.

As the coronal alignment changed from varus to valgus, the femoral external rotation increased (r = 0.217; p < 0.0001). The mean (SD) CTA was 5.42° (1.45°) in the varus group, 5.71° (1.45°) in the neutral group, and 6.37° (1.58°) in the valgus group. For every 1° in coronal alignment increment from varus to valgus, there was a 0.05° increment of the CTA. No correlation was found between the global coronal alignment (HKA) and the FV (r = 0.074; p = 0.148). However, there was a weak positive correlation between the FV and the sFLDA (r = 0.128; p = 0.012). The mean (SD) FV was 10.78° (7.4°) in the varus group, 10.7° (8°) in the neutral group, and 12.14° (8.75°) in the valgus group. As the coronal alignment changed from varus to valgus, the external TT increased (r = 0.248; p < 0.0001). The mean (SD) TT was 18.59° (9.7°) in the varus group, 23.04° (9.08°) in the neutral group, and 24.61° (10.4°) in the valgus group. The comparison of the rotational values depending on the varus, neutral, or valgus axis is shown in Fig. 2. We have performed a multivariable linear regression to study the effect of sFLDA, PTMA, CTA, FV, and TT on the dependent variable (HKA). 50.2% of the variance in HKA is due to sFLDA, 27.6% due to PTMA. There has been a minimal influence of posterior condylar angle (PCA) (0.6%) and TT (0.5%). PCA is defined as the angle between the PCL and the surgical transepicondylar axis (sTEA) (a line connecting the most prominent point of the lateral epicondyle with the lowest point of the medial sulcus). Scatter plots of rotational values concerning coronal alignment are shown in Fig. 3. The multivariate regression analysis of the rotational variables did not show an influence to be considered of the independent variables analyzed. We found significant differences by gender, as shown in Table 2. Females presented significantly (p < 0.0001) higher femoral external rotation (CTA), femoral anteversion (FV), and TT, compared to males (Table 3). Cohen’s effect size d was calculated for all results.

**Table 2:** Coronal and rotational parameters according to gender.

	Female cases (n = 224)	Male cases (n = 161)	p-value	d-value
HKA (°)	177.38 (6.24)	174.74 (5.25)	< 0.0005	2.639
sFDLA (°)	92.69 (2.82)	90.74 (2.54)	< 0.0005	1.959
PTMA (°)	87.42 (2.68)	87 (3.08)	0.167	0.408
CTA (°)	5.85 (1.53)	5.29 (1.39)	< 0.0005	0.559
FV (°)	12.3 (7.5)	9.07 (7.64)	< 0.0005	3.224
TT (°)	22.2 (9.96)	17.79 (9.4)	< 0.0005	4.412

Females differed significantly from males in the hip-knee-ankle angle and in the supplementary angle of the femoral lateral distal angle and presented higher femoral external rotation, femoral anteversion and tibial external rotation (independent two-sample t-test). Angular values expressed as the mean and (standard deviation). CTA, condylar twist angle. FV, Femoral Version measured between the femoral neck axis and the posterior condylar line. HKA, hip-knee-ankle angle. PTMA, proximal medial tibial angle. sFDLA, supplementary angle of the femoral lateral distal angle. TT, Tibial Torsion. d-value to qualify the magnitude of an effect (the difference between means) can be interpreted according to the criteria by Hopkins et al. ^21^: less than 0.2, trivial; 0.2 to 0.59, small; 0.6 to 1.19, moderate; 1.20 to 2, large; 2 to 3.99, very large, and greater than 4, extremely large.

**Table 3:** Rotational parameters according to gender and to the coronal alignment group.

	Varus group (HKA < 177°)				Neutral group (HKA 177° to 183°)				Valgus group (HKA > 183°)			
	Female	Male	p-value	d-value	Female	Male	p-value	d-value	Female	Male	p-value	d-value
CTA (°)	5.66 (1.55)	5.17 (1.28)	0.007	0.492	5.82 (1.3)	5.48 (1.71)	0.32	0.342	6.49 (1.6)	6 (1.47)	0.337	0.487
FV (°)	12.35 (7.28)	9.1 (7.19)	< 0.0005	3.247	10.97 (7.29)	10.15 (9.4)	0.665	0.824	13.94 (8.35)	6.61 (7.83)	0.007	7.322
TT (°)	19.98 (9.96)	17.09 (9.22)	0.018	2.886	23.77 (9.42)	21.59 (8.34)	0.312	2.175	27.3 (8.47)	16.35 (11.73)	0.001	10.453

Femoral external rotation in our study is significantly higher in females than in males only in the varus coronal alignment group. Femoral anteversion and tibial external torsion are significantly higher in females than males in varus and valgus alignments, but not in neutral alignment (independent two-sample t-test). Angular values expressed as the mean and (standard deviation). *CTA* condylar twist angle, *FV* Femoral Version measured between the femoral neck axis and the posterior condylar line, *HKA* hip-knee-ankle angle, *TT* Tibial Torsion. d-value to qualify the magnitude of an effect (the difference between means) can be interpreted according to the criteria by Hopkins et al.^21^: less than 0.2, trivial; 0.2–0.59, small; 0.6–1.19, moderate; 1.20–2, large; 2–3.99, very large, and greater than 4, extremely large.

## Discussion

This study was set up to investigate the relationship among the coronal alignment and the rotational profile of the lower extremities, in the Caucasian population, with knee OA. The most important finding of the present study was that in the examined population, the external femoral rotation (CTA) and the tibial external torsion increase as the valgus alignment increases. In contrast, there was no significant correlation between the coronal alignment and the degree of femoral ante- or retro-version.

There is no consensus on the optimal angle in CT-scan studies to describe femoral rotation, either pre- or post-operatively. Some authors^9,22–26^ choose to use the PCA. Others prefer to use the CTA^10,11,18,27–32^. Furthermore, others have employed and analyzed the advantages and disadvantages of both PCA and CTA^7,27,28,33–36^. In our measurements, we have used the CTA. The most salient point of the medial epicondyle, required for the CTA, is easier to identify than the medial sulcus needed for the PCA^27,28,34–36^.

It has been widely published that femoral component rotational misalignment leads to an unsatisfactory outcome, including discomfort, pain, patella maltracking, instability, stiffness, inadequate kinematics during gait, arthrofibrosis, and might decrease the survival of the prosthesis^31,32,37^. More controversial and still an unanswered question is the femoral component's optimal rotation to avoid such unsatisfactory results^37–41^. Multiple reports in the literature evaluate the most common techniques used to adjust the femoral component axial alignment with contradictory conclusions. In the measured resection technique bony landmarks are used as references to set femoral rotation by aligning the flexion–extension axis parallel to the sTEA, perpendicular to the anteroposterior axis of the trochlear groove (Whiteside's line), or 3° externally rotated to the PCL (based on multiple papers the PCL is on average 3° internally rotated relative to the sTEA). In the gap-balancing technique, the femoral component is positioned parallel to the resected proximal tibia, with both collateral ligaments equally tensioned. The kinematic alignment introduces a different rotational concept since it aims at the geometrical center axis as the flexion–extension axis of the TKA. An exact correlation between the rotational placement of the femoral component and the clinical and functional outcome has not yet been demonstrated, which underlines the importance of each patient's individuality^41^.

Regardless of the angle chosen to evaluate the femoral rotation, different reports have shown a correlation between the coronal alignment and the rotational geometry of the distal femur. Femoral external rotation increases as coronal alignment changes from varus to valgus^7,9–11,35^. To the best of our knowledge, Aglietti et al. first reported a linear relationship between the coronal alignment and the PCA^35^. Luyckx et al.^7^ published a clear linear relationship between the overall coronal alignment and the rotational geometry of the distal femur (for every 1° in coronal alignment increment from varus to valgus, there is a 0.1° increment in PCA) consistent with Aglietti's prior postulate (a 1° PCA increment was observed with every 10° increment of coronal deformity from varus to valgus)^35^. In our study, a 0.05° CTA increment was observed for every 1° increment of coronal deformity from varus to valgus. On the other hand, Aglietti et al. reported similar femoral external rotation (assessed by PCA values) for women and men. In our study, we observed a significant difference, with higher femoral external rotation (assessed by CTA values) in women, when considering the 385 cases of the study and when analyzing the cases with varus coronal alignment. The values of femoral anteversion and external tibial torsion were significantly higher in women than in men (except for the comparison of neutral coronal alignment cases).

The relationship between coronal alignment and femoral version has not been clearly defined, and results from different publications are inconsistent. In the study by Puthumanapully et al.^42^, the femoral neck measured to the PCL was significantly less anteverted for varus knees compared with neutral knees, and a weak positive correlation was found between varus angle and femoral neck version in the varus cases. On the other hand, in the articles by Lim et al.^43^ (using the PCL) and Chang et al.^11^ (using the cTEA), no correlation was detected between the coronal alignment and the degree of femoral version. Chang et al.^11^ observed that the degree of femoral anteversion (measured using the PCL), increased as the coronal alignment changed from varus to valgus. In the present study, no relationship between coronal alignment and femoral version has been detected.

In line with other studies^11^, the degree of tibial external torsion (rotation of the tibia along its longitudinal axis causes external rotation of the distal tibial relative to the proximal) increased as the coronal alignment changed from varus to valgus in our case. The range of tibial torsion is extensive in the general population, and the difficulty of achieving the tibial component's neutral alignment using a fixed anatomical reference at the ankle joint in each patient has been described^44^. The relationship between tibial torsion and coronal alignment remains one of the least studied topics in TKA surgery.

Knee anatomic differences across ethnic groups are thoroughly discussed in the literature^10,12–16^. Murgier et al.^10^ confirm the difference of the CTA value (which they call cPCA) between Asians (6.4° ± 2.59°) and Caucasians (5.5° ± 2.36°) free of knee OA. We have determined the same parameters published by Chang et al. (422 cases of Asian patients with knee OA evaluated with CT-scan)^11^. As previously stated, we have also found a positive correlation between coronal alignment and external femoral rotation (measured as CTA) and between coronal alignment and external TT. We have not established any relationship of the femoral version with the coronal alignment. The differences in results between Chang's study^11^ and ours are shown in Table 4. There seems to be an increased external femoral rotation, and an increased external tibial torsion in Asians compared to Caucasians. This is consistent with the previous results of other authors^9,10^ and is of clinical importance, indicating that individual rotation determination is necessary.

**Table 4:** Coronal and rotational parameters according to ethnic groups.

	CTA (°)			FV (°)			TT (°)		
	Varus	Neutral	Valgus	Varus	Neutral	Valgus	Varus	Neutral	Valgus
Chang et al.^11^	6.6 (4.8)	7.4 (2.5)	10.2 (1.9)	10.9 (7)	12.1 (6)	16.7 (5.8)	22.6 (7.2)	26.3 (6.9)	32.6 (6.2)
León-Muñoz et al.	5.42 (1.45)	5.71 (1.45)	6.37 (1.58)	10.78 (7.4)	10.7 (8)	12.14 (8.75)	18.59 (9.7)	23.04 (9.08)	24.61 (10.4)

Chang et al.^11^ evaluated with CT-scan 422 cases of Asian patients with knee OA. We have evaluated 385 CT-scan based 3D virtual models of Caucasians patients with knee OA. In both studies, the patients have been divided into three groups according to the coronal alignment (HKA < 177° = varus; HKA 177° to 183° = neutral and HKA > 183° = valgus). Angular values expressed as the mean and (standard deviation). *CTA* condylar twist angle, *FV* Femoral Version measured between the femoral neck axis and the posterior condylar line, *TT* Tibial Torsion.

There are some limitations to our study. Firstly, the measurements were performed by a single observer, and we did not determine the inter-observer bias. However, in a previous study^9^ with trained Medacta planning engineers, the inter-observer variability on CT-scan and 3D models was determined on five different operators. It gave a standard deviation of 0.5°, and thus, inter-observer bias was assumed to be minimal. In the same study^9^, the intra-observer variability was analyzed on 15 samples and gave a standard deviation of 0.7°. Secondly, the coronal alignment measurements were performed on the CT-scan based 3D model and, therefore, under non-weight-bearing conditions. Significant discrepancies exist among weight-bearing full-length anteroposterior radiographs of the lower limb (LLRs) and supine non-weight-bearing CT-scan images, MRI images, or 3D virtual models in the assessment of the coronal alignment, despite an adequate correlation^17,45,46^. We preferred for our study the coronal measurements from the CT-scan-based models. Despite the standardized conduct of LLR studies in our institution, we have not controlled rotation by employing, for example, the relative position of the proximal fibula to the proximal tibia^47^. We thought, therefore, that using LLRs measurements would detract from accuracy. Measuring the mechanical alignment in the models has the advantage of exclusively analyzing the bone profile and having no influence on cartilage wear or potential soft tissue laxity. On the other hand, it has the disadvantage of not reflecting the influence on the alignment of soft tissue conditions or the degree of cartilage wear and how it affects rotational characteristics. Thirdly, assessing the rotation profile of the lower limb using 3D CT-scan based models is a static method that does not provide scope for the study of kinematic and kinetic parameters such as gait analysis. Finally, in comparison with the results of Chang et al. (evaluating Asian patients), we have to consider the bias of their measurements on CT-scans and ours on 3D models. However, we think, as published by Buck et al.^48^ in an analysis of 3D models obtained from biplanar radiographs, that femoral and tibial torsion measurements on 3D models are interchangeable with standard CT-scan measures in patients with OA of the knee.

In summary, by employing mechanical alignment concepts, individual rotation determination is necessary; in particular, patients with femoral valgus may need more femoral external rotation. A linear relationship was observed between the coronal alignment and the rotational geometry of the distal femur. This correlation also occurs with the tibial torsion. It will be interesting to establish the influence for successful surgery of an individualized pre-operative assessment of the lower extremity rotational patterns in patients undergoing TKA, considering that the outcomes also depend on factors other than mechanical (e.g., co-morbidities, psychological, behavioral, and social factors).
